# Enhancing High-Pressure Capillary Rheometer Viscosity Data Calculation with the Propagation of Uncertainties for Subsequent Cross-Williams, Landel, and Ferry (WLF) Parameter Fitting

**DOI:** 10.3390/polym15143147

**Published:** 2023-07-24

**Authors:** Martin Hubmann, Stephan Schuschnigg, Ivica Ðuretek, Jonas Groten, Clemens Holzer

**Affiliations:** 1 Polymer Processing, Department of Polymer Engineering and Science, Montanuniversitaet Leoben, 8700 Leoben, Austria; 2 Joanneum Research Forschungsgesellschaft mbH, Franz-Pichler Str. 30, 8160 Weiz, Austria

**Keywords:** viscosity measurement, viscosity evaluation, shear rate dependency, pressure-dependency, Cross-WLF, polycarbonate, PC, simulation

## Abstract

Measuring the shear viscosity of polymeric melts is an extensive effort frequently performed in high-pressure capillary rheometers, where the pressures required to push the melt through a capillary at various temperatures and volumetric flow rates are recorded. Then, the viscosity values are obtained through Bagley and Weissenberg–Rabinowitsch corrections involving parameter fitting. However, uncertainties in those conversions due to pressure variations and measurement inaccuracies (random errors) affect the accuracy of the consequently calculated viscosities. This paper proposes quantifying them through a propagation of uncertainties calculation. This has been experimentally demonstrated for a polycarbonate melt. In addition, the derived viscosity uncertainties were used for the weighted residual sum of squares parameter estimation of the Cross-WLF viscosity model and compared with the coefficients obtained using the standard residual sum of squares minimization approach. The motivation was that, by comparison, individual poorly measured viscosity values should have a less negative impact on the overall fit quality of the former. For validation, the rheometer measurements were numerically simulated with both fits. The simulations based on the Cross-WLF fit, including the derived viscosity uncertainties, matched the measured pressures ~16% more closely for shear rates below 1500 1/s. Considering the uncertainties led to more precise coefficients. However, both fits showed substantial deviations at higher shear rates, probably due to substantial non-isothermal flow conditions that prevailed during these measurements. A capillary rheometer experiment was also simulated using arbitrarily chosen Cross-WLF parameters to exclude such systematic errors. A normally distributed error was then applied to the simulated pressures before re-fitting the parameters. Again, taking advantage of the derived viscosity uncertainties, the fit could recover the initial parameters better.

## 1. Introduction

With ever-increasing demands in part complexities, computational fluid dynamics have become a must-have tool to investigate the processability of plastic parts even before manufacturing the processing tools. Consequently, multiple material properties must be carefully characterized (shear viscosity, pvT-behavior, specific heat capacity, thermal conductivity) [1]. Arguably among the most important is the polymer fluid’s shear viscosity

(1) $$\eta =\frac{\tau }{\dot{\gamma }},$$

which is the resistance to deformation in a shear flow, defined as the quotient of shear stress τ and shear rate $\dot{\gamma }$. A high viscosity η will result in a high-pressure demand p during manufacturing, such as extrusion or injection molding, that can impede successful production. The viscosity of most (thermo)plastic melts depends on the temperature T, the shear rate, and, to some extent, the pressure p [2,3].

**Figure 1:**
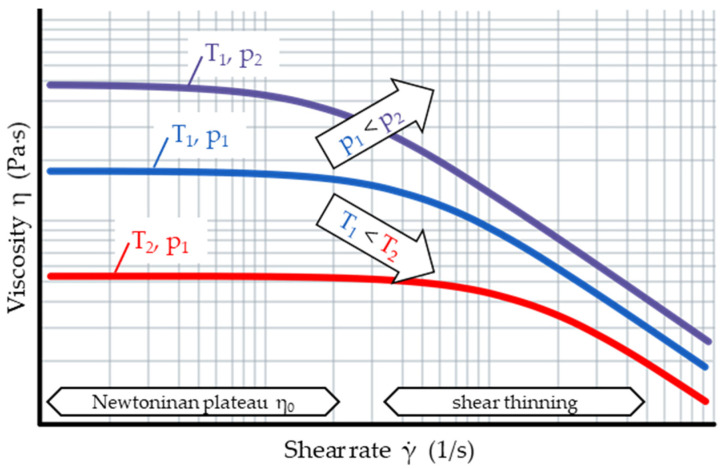
Many polymeric melts exhibit a Newtonian viscosity plateau at low and shear thinning behavior at high shear rates. The viscosity decreases with increasing temperature and decreasing pressure.

Figure 1 shows a typical viscosity curve of thermoplastics: A Newtonian plateau is present at low shear rates, transitioning into a shear thinning domain with increasing shear rates.

One of the most used viscosity models in simulation packages is the seven-parameter Cross-Williams, Landel, and Ferry (Cross-WLF) model. It can describe the viscosity in function of the shear rate, including the Newtonian plateau and shear thinning behavior (Cross law) and of the temperature and the pressure (WLF) [4,5,6,7,8]. It reads

(2) $$\eta \left(\dot{\gamma },T,p\right)=\frac{D_{1}\cdot e^{\frac{-A_{1}\cdot \left({T\text{-}D}_{2}{\text{-}D}_{3}\cdot p\right)}{A_{3}{+T\text{-}D}_{2}}}}{1+{\left(\frac{D_{1}\cdot e^{\frac{-A_{1}\cdot \left({T\text{-}D}_{2}{\text{-}D}_{3}\cdot p\right)}{A_{3}{+T\text{-}D}_{2}}}\cdot \dot{\gamma }}{\tau^{*}}\right)}^{1\text{-}n}},$$

with its parameters explained in Table 1.

In practice, $D_{1}$, $\tau^{*}$, n, $A_{1}$, $A_{3}$, and $D_{3}$ are determined via curve fitting while setting $A_{3}=51.6$ K and $D_{2}=T_{g}$ (polymer’s glass transition temperature) [9]. Commonly, the least squares method is used to minimize the residual sum of squares

(3) $$\mathrm{RSS}=\sum_{i=1}^{n}\left({\left[f\left(x_{i};\;b\right)-y_{i}\right]}^{2}\right)\to \;\underset{b}{\min}\mathrm{RSS}\left(b\right),$$

where f designates the model function, in our case, the Cross-WLF model with its independent variables $x_{i}$ ($\dot{\gamma }$, T, and p) and parameters b (n, $A_{1}$, …) and dependent variables $y_{i}$ designating the measured data (η). In practice, numerical algorithms are used to find the values of the parameters b so that the RSS (Equation (3)) is minimized [10,11].

Some simulation software (such as Autodesk Modflow Insight or Cadmould [4,12]) limit the range in which they accept the coefficients to preserve their physical “meaningfulness” (compare Table 1). For instance, limiting the power index n ≥ 0 guarantees that a lower temperature always yields a higher viscosity (at a given shear rate and pressure).

Naturally, to fit the Cross-WLF parameters, the viscosity must be measured first. Frequently, this is performed with high-pressure capillary rheometers (HPCRs). Those devices are suitable for determining the shear viscosity within the relevant shear rate range to numerous processing techniques. If needed, for lower shear rates ($\dot{\gamma }⪅\;100\;1/s$), additional rotational rheometry (frequently cone-plate or plate-plate rheometers in oscillatory mode) can be performed.

**Figure 2:**
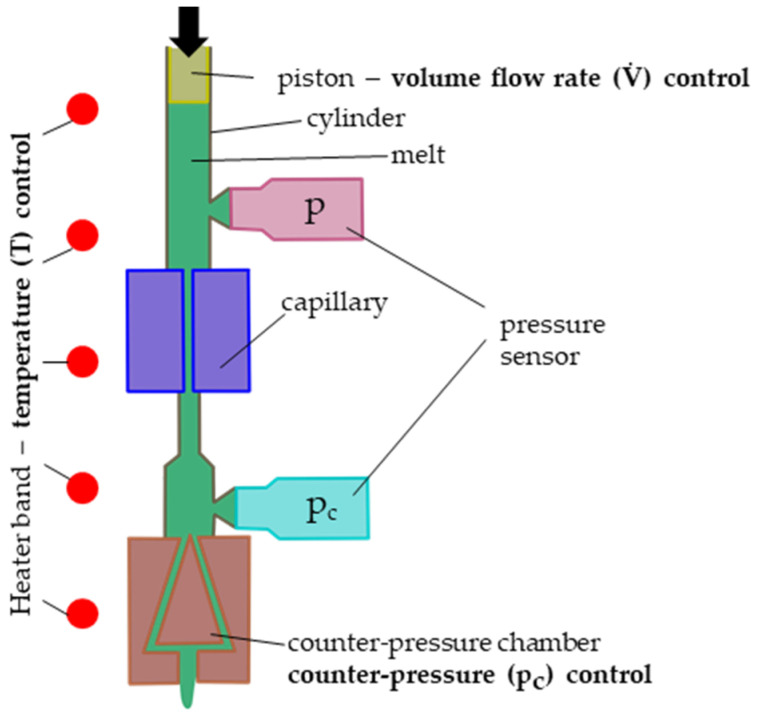
Schematic of a high-pressure capillary rheometer (HPCR).

The basic concept of an HPCR measurement is to derive the viscosity of a polymeric melt from the pressure drop caused when it is pushed by the controlled movement of the piston at a defined volumetric flow rate through a round capillary. Figure 2 shows a schematic of a HPCR, where the melt is heated in a cylinder.

For the HPCR experiment and subsequent viscosity calculation, the following assumptions and premises are required [13]:

- Laminar flow;
- Isothermal conditions;
- Wall adherend melt;
- Incompressible melt;
- Non-pressure-dependent melt;
- Stationary conditions;
- Newtonian fluid [14].

Heater bands bring the melt and capillary (wall) to the selected temperature T. Then, a piston pushes the melt through the capillary with diameter d at a defined volumetric flow rate $\dot{V}$, resulting in an apparent wall shear rate

(4) $${\dot{\gamma }}_{a}=\frac{32\cdot \dot{V}}{\pi \cdot d^{3}}.$$

**Figure 3:**
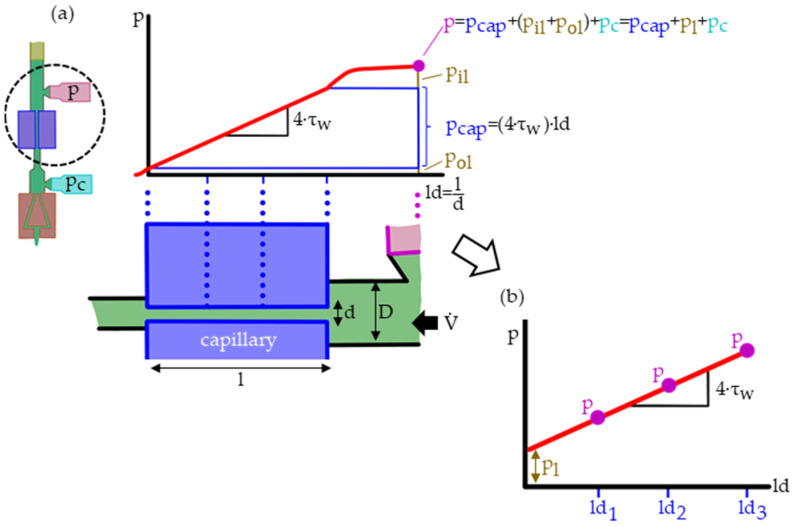
Schematic of the presumed pressure drop within a high-pressure capillary rheometer (HPCR): the measured pressure p comprised within the capillary linearly decreasing pressure $p_{\mathrm{cap}}$ and the pressure losses $p_{l}$ (**a**). Those pressure losses $p_{l}$ can be deduced, and the wall shear rate $\tau_{w}$ calculated when measuring with multiple capillaries of different ld ratios and plotting them in Bagley correction (BC) plots (**b**).

The wall shear stress in the capillary is

(5) $$\tau_{w}=\frac{p_{\mathrm{cap}}}{4\cdot \mathrm{ld}},$$

where $p_{\mathrm{cap}}$ is the (linear) pressure drop within the capillary, and ld is the ratio

(6) $$\mathrm{ld}=\frac{l}{d},$$

of the capillary of length l and diameter d [14,15]. Due to the narrow capillary diameter (typically d=0.5–1 mm), it is impossible to directly mount a pressure sensor. Thus, the pressure sensor must be located at the cylinder (typically diameter D=12 mm). Therefore, the measured pressure

(7) $${p=p}_{\mathrm{cap}}+p_{l}=p_{\mathrm{cap}}+(p_{\mathrm{il}}+p_{\mathrm{ol}})$$

comprises the pressure drop within the capillary $p_{\mathrm{cap}}$ and an additional pressure loss $p_{l}$. This loss consists of the inlet pressure loss $p_{\mathrm{il}}$—between the sensor and the capillary—and the lesser outlet pressure losses $p_{\mathrm{ol}}$ after the capillary. Figure 3a illustrates the pressure drop between the pressure sensor and the capillary exit.

The pressure losses $p_{l}$ are assumed independent of the capillary’s length. Consequently, they can be subtracted through Bagley corrections (BCs) by measuring with differently long capillaries and extrapolating to a die length of zero, as illustrated in Figure 3b [16].

**Figure 4:**
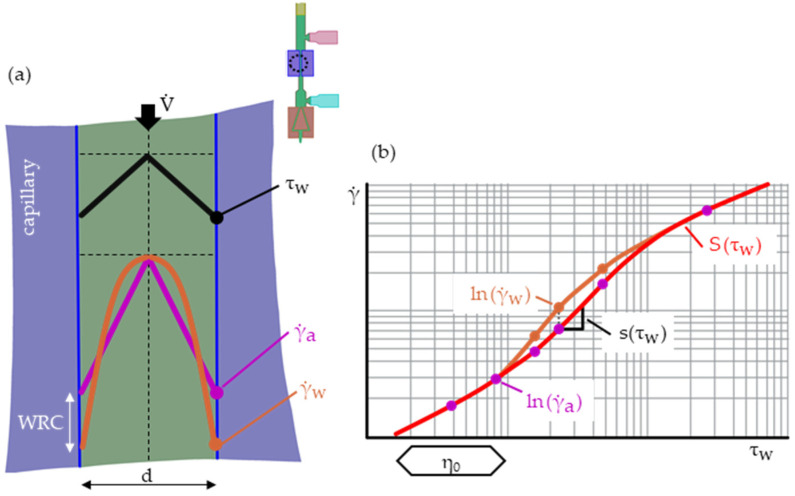
Schematic of the radial shear stress τ and shear rate $\dot{\gamma }$ profile in a HPCR (**a**). The WRC converts the apparent wall shear rate ${\dot{\gamma }}_{a}$ into the true wall shear rate ${\dot{\gamma }}_{w}$ (non-Newtonian fluids), which is performed through the WRC plots and fitting polynomials (**b**) [17,18].

Next, the apparent wall shear rate ${\dot{\gamma }}_{a}$ has to be corrected to the true wall shear rate ${\dot{\gamma }}_{w}$ for non-Newtonian fluid behaviors (Figure 4a). This is performed with the Weissenberg–Rabinowitsch correction (WRC) plots, as illustrated in Figure 4b [17].

The WRC for (round) capillaries reads

(8) $${\dot{\gamma }}_{w}=\frac{{\dot{\gamma }}_{a}}{4}\cdot \left(3+s\left(\tau_{w}\right)\right).$$

Here, $s\left(\tau_{w}\right)$ is the slope

(9) $$s\left(\tau_{w}\right)=\frac{\mathrm{dS}\left(\tau_{w}\right)}{\mathrm{dln}\left(\tau_{w}\right)}$$

in the double logarithmic apparent shear rate vs. wall shear stress graph (compare Figure 4b). It is obtained by fitting a polynomial

(10) $$S\left(\tau_{w}\right)=\ln\left({\dot{\gamma }}_{a}\left(\tau_{w}\right)\right)=\sum_{i=0}^{\mathrm{dg}}\left(a_{i}\cdot \ln{\left(\tau_{w}\right)}^{\mathrm{dg}-i}\right),$$

where $a_{i}$ are the fitting parameters (coefficients). WRC polynomials are usually of second or third degree (dg).

Finally, the hence-derived shear stress $\tau_{w}$ and shear rate ${\dot{\gamma }}_{w}$ yield the true viscosity η (at the wall) through Equation (1).

In some HPCR configurations, a counter-pressure chamber with a valve and a second pressure sensor $p_{c}$ might be added (see Figure 2) to elevate the pressure level

(11) $${p=p}_{\mathrm{cap}}+p_{c}+p_{l}.$$

This additional measurement effort is necessary to quantify the pressure dependency of the melt’s viscosity. For some polymers, such as polycarbonate (PC), it is vital to determine the pressure dependence (coefficient $D_{3}$ in Equation (2) of the Cross-WLF model) for accurate process simulations [6,19,20].

To different extents, none of the above-listed premises are fully fulfilled. Many studies in the literature have addressed the different challenges and limitations of the HPCR measurement. For instance, the fact that the viscous dissipation of the melt becomes (more) critical with increasing shear rates is well documented [14,21,22,23]. Here, a non-uniform temperature profile develops, resulting in a hot boundary layer of low viscosity (more plug flow-like). A downward curvature within the BC plots can result from the viscous dissipation as it becomes more pronounced with increasing capillary length.

However, in contrast, BC plots with an upwards curvature, can occur if the pressure dependency of the melt is non-neglectable. In such cases, BC polynomials of second degree are more suitable to estimate the pressure losses $p_{l}$ [15,24,25].

Some strategies for treating viscous dissipation, pressure dependency, partial wall slip, and the compressibility of melts were developed by Laun [14,23]. Malkin et al. proposed calculating the pressure loss in short dies through the polymer’s average flow and relaxation times [26].

A measure for the severity of the viscous heating is the Cameron number, the division of the heat conduction normal to the flow through the convective heat transport

(12) $$\mathrm{Ca}=\frac{\lambda \cdot 4\cdot L}{\rho \cdot c_{p}\cdot \dot{V}\cdot d^{2}},$$

where λ is the thermal conductivity, ρ the density, and $c_{p}$ the specific heat capacity. The flow is in the thermal equilibrium regime for Ca > 1 and within the adiabatic regime for Ca < 0.01, with a transition regime between them [27].

As summarized above, many authors [14,15,21,22,23,26] have performed comprehensive work to reduce the negative impacts of systematic errors such as viscous dissipation on the HPCR measurements. However, to our knowledge, no publication in the literature studied and calculated the uncertainties in the viscosity data due to random errors. In practice, if the resin is difficult to measure for various reasons, the results are poorly reproducible (pressure) readings. Especially then, not only the viscosity value but also its uncertainty, for example, quantified in terms of its standard deviation ${\eta \pm u}_{\eta }$ should be reported.

In this work, we estimate the viscosity uncertainties $u_{\eta }$ that occur during HPCR measurements and subsequent data processing (BC, WRC) by performing a propagation of uncertainty (PoU, or propagation of error) calculation. The generalized PoU reads

(13) $$u_{y}=\sqrt{\mathrm{grad}(y^{T})\cdot V_{x}\cdot \mathrm{grad}(y)},$$

with covariance matrix $V_{x}$ (a measure of the correlated uncertainties in the input data) and function y [28]. PoU calculations are performed when the required quantities (y) cannot be directly measured but are the consequence of measured values (${x\pm u}_{x}$) that are transformed through a series of calculations (y=f(x)). Consequently, uncertainties in those measured values ($u_{x}$), expressed in the generalized case by the covariance matrix ($V_{x}$) propagate via the functions into the calculated values (${y\pm u}_{y}$). In the present case, x denotes the measured pressures, y denotes the viscosities, and $V_{x}$ denotes the results from the BC and WRC parameter fittings.

In practice, frequently the relative uncertainty

(14) $$\delta_{y}{=u}_{y}/y$$

is used, which is the ratio of the value’s uncertainty $u_{y}$ to the magnitude of the value y.

In the following, the viscosity uncertainties $u_{\eta }$ can be used to estimate the Cross-WLF parameters in weighted residual sum of squares (WRSS) optimizations. The corresponding minimization function reads

(15) WRSS=∑i=1n(1uyi2·[fi(xi; b)-yi]2)→ minbS(b),

which resembles the RSS (Equation (3)) modified by the expression 1uyi2 [10,11]. Consequently, the fit quality should improve because the knowledge of less reliably measured viscosity values (larger $u_{\eta }$) is incorporated into the regression.

**Figure 5:**
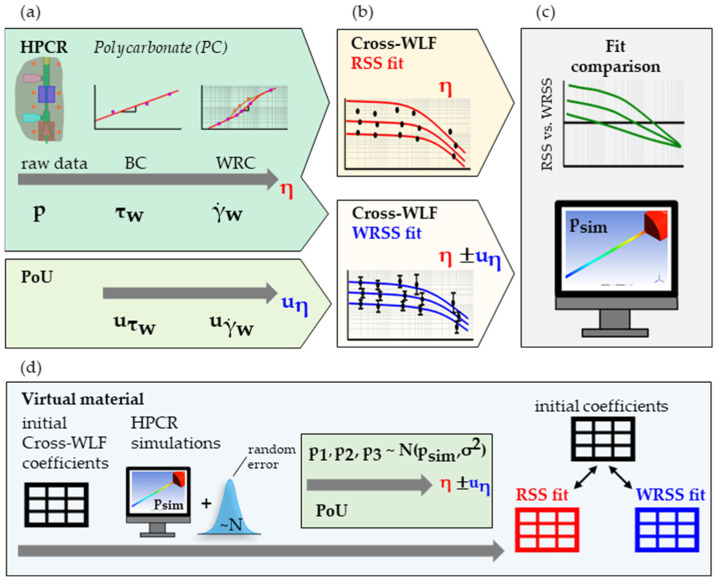
Schematic of the presented approach: The viscosities η of a PC were measured using a HPCR, with a PoU performed alongside to estimate the viscosity uncertainties $u_{\eta }$. (**a**). Next, two sets of Cross-WLF parameters were fitted, minimizing the RSS or the WRSS (**b**). Finally, the two fits were compared, and simulations for benchmarking were performed (**c**). Similarly, the HPCR simulations of a virtual material with fabricated Cross-WLF parameters were performed, and the resulting pressures were “repeated” by subjecting them to a normally distributed error. Then, the Cross-WLF parameters were re-fitted through the RSS and WRSS (based on the postulated PoU calculation) approaches (**d**).

For validation, the viscosity of a PC was measured in an HPCR (Figure 5a), and two sets of Cross-WLF parameters were fitted. One minimizes the standard RSS (Equation (3)), and the other minimizes the WRSS (Equation (15)) utilizing the viscosity uncertainties $u_{\eta }$ derived in our proposed PoU (Figure 5b). Numerical flow simulations of the HPCR capillary with both sets of Cross-WLF coefficients were made and benchmarked (Figure 5c).

In addition, further scrutinizing the proposed method, the HPCR simulation model and a “virtual material” using fabricated Cross-WLF parameters were used to virtually replicate a HPCR measurement. First, the simulated pressures were “repeated” by subjecting them to a normally distributed error. Next, they were used to re-fit the initial Cross-WLF parameters through the RSS and WRSS (using the weights from the PoU calculation) method. Thus, any potential systematic error in the HPCR measurement was strictly excluded, and only the influence of the random errors could be studied.

## 2. Materials and Methods

### 2.1. Polycarbonate Lexan OQ 1028

The polycarbonate PC Lexan OQ1028 (Sabic, Riyadh, Saudi Arabia) was used in this study. Its specific heat capacity and thermal conductivity in the molten state at 325 °C were measured at $c_{p}=1920\;J/(\mathrm{kg}\cdot K)$, and λ=0.25 W/(m·K), respectively. These were determined using a differential scanning calorimeter DSC1 (Mettler Toledo GmbH, Greifensee, Switzerland) at a cooling rate of −20 K/min (ISO 11357) and a K-System II (Advanced CAE Technology Inc., Ithaca, NY, USA) based on line-source method (ASTM D5930-17), respectively. The density $\rho ={1.09\;g/\mathrm{cm}}^{3}$ (at 325 °C and 0 bar) was derived from the Tait pvT coefficients provided by the supplier. According to the manufacturer, the melt flow rate (MFR) of the Lexan OQ1028 is 10 g/10 min (250 °C, 1.2 kg, ASTM D 1238) [29].

### 2.2. High-Pressure Capillary Rheometer (HPCR)

A Göttfert Rheograph 2002 (GÖTTFERT Werkstoff-Prüfmaschinen GmbH, Buchen, Germany) HPCR equipped with a barrel D=12 mm in diameter, to which a counter-pressure chamber (by GÖTTFERT) could be installed, was used. In addition, several calibrated Gefran M30 (Gefran SpA, Provaglio d’Iseo, Italy) pressure sensors of different pressure ranges were utilized. The sensor was positioned in the barrel 15 mm above the capillaries entry (with an entry angle of 180°).

Table 2 breaks down the test plan. Three temperature settings, two counter-pressure levels, and measurements without counter-pressures were investigated using capillaries of a diameter of d=1 mm and length-to-diameter ratios of ld=10, 20, and 30. In addition, capillaries of diameter d=0.5 mm (ld = 10, 20, and 30) were used without counter-pressure for measurements at higher shear rates.

Each measurement was performed n=3 times, and the pressures were recorded. The HPCR cylinder was manually filled with the PC granulates bit by bit to firmly compress the molten layers and remove trapped air. Afterward, the piston was lowered for contact with the polymer, and a melting/waiting time of 4 min was abided to ensure a homogenous melt at the selected temperature T. Finally, the resulting pressures p was predefined, and in ascending order, sorted the apparent shear rates ${\dot{\gamma }}_{a}$ which were recorded once the pressures had stabilized.

### 2.3. Viscosity Calculation Derivation

This section describes the performed HPCR pressure raw data conversion to obtain the viscosities with related uncertainties ${\eta \pm u}_{\eta }$. Possible fluctuations in the input parameters, like temperature, volumetric flow rate, counter-pressure, and material homogeneity, affect the measured pressures. Consequently, those random pressure variations are reflected in the uncertainties of the derived viscosities.

#### 2.3.1. Bagley Correction (BC) and Wall Shear Stress

The wall shear stresses $\tau_{w}$ were obtained through linear BCs. Individual BCs were made for each set of apparent shear rate ${\dot{\gamma }}_{a}$, at the different temperatures T and counter-pressures $p_{c}$: here, the recorded pressures p obtained using the three capillary ratios ld are presumed to follow the relation

(16) $${p=p}_{\mathrm{cap}}+p_{c}+p_{l}=\left(b_{0}\cdot \mathrm{ld}\right)+p_{c}+p_{l}$$

(deduced from Equations (6), (7) and (11)). The parameters $b_{0}$ and $p_{l}$ (pressure loss term) and their standard deviation errors ($u_{b_{0}}$ and $u_{p_{l}}$) were estimated using the Python scipy.optimize.curve_fit [11] routine (minimizing RSS of Equation (3)).

The violations to the measurement presumptions can lead to a negative pressure loss when extrapolated to a die with ld=0. This is physically untenable. Thus, the pressure loss was restricted to $p_{l}\;\geq \;0$ in the parameter fitting to avoid artificially inflating the $b_{0}$ coefficient, which yields, according to Equation (5), the wall shear stress

(17) $$\tau_{w}=\frac{b_{0}}{4}.$$

The related uncertainty is then given by

(18) $$u_{\tau_{w}}=\sqrt{{\left(\frac{\partial \tau_{w}}{\partial b_{0}}\cdot u_{b_{0}}\right)}^{2}\;}=\sqrt{{\left(\frac{1}{4}\cdot u_{b_{0}}\right)}^{2}}=\frac{u_{b_{0}}}{4}$$

(applying Equation (13)).

#### 2.3.2. Weissenberg–Rabinowitsch Correction (WRC) and Wall Shear Rate

The wall shear rates ${\dot{\gamma }}_{w}$ were obtained through WRCs. Individual third-degree WRCs polynomials

(19) $$S\left(\tau_{w}\right)=\ln\left({\dot{\gamma }}_{a}\left(\tau_{w}\right)\right)=\sum_{i=0}^{3}\left(a_{i}\cdot \ln{\left(\tau_{w}\right)}^{3\text{-}i}\right)$$

were fitted for each tested temperature T and counter-pressure $p_{c}$ combination. The parameters $a_{0}$, $a_{1}$, $a_{2}$, and $a_{3}$ and the corresponding covariance

(20) $$V_{a}=\left[\begin{array}{cccc} u_{a_{00}}{}^{2} & u_{a_{01}}{}^{2} & u_{a_{02}}{}^{2} & u_{a_{03}}{}^{2}\\ u_{a_{10}}{}^{2} & u_{a_{11}}{}^{2} & u_{a_{12}}{}^{2} & u_{a_{13}}{}^{2}\\ u_{a_{20}}{}^{2} & u_{a_{21}}{}^{2} & u_{a_{22}}{}^{2} & u_{a_{23}}{}^{2}\\ u_{a_{30}}{}^{2} & u_{a_{31}}{}^{2} & u_{a_{32}}{}^{2} & u_{a_{33}}{}^{2} \end{array}\right]$$

were estimated through the numeric least square method scipy.optimize.curve_fit [11]. Using Equations (8) and (9) yields the wall shear rate

(21) $${\dot{\gamma }}_{w}=\frac{{\dot{\gamma }}_{a}}{4}\cdot \left(3+\left(3\cdot a_{0}\cdot {\ln(\tau }_{w}{)}^{2}+2\cdot a_{1}\cdot {\ln(\tau }_{w})+a_{2}\right)\right).$$

#### 2.3.3. Viscosity Calculation

Equations (1), (8) and (9) finally yield the (wall) viscosity

(22) $$\eta =\frac{\tau_{w}}{{\dot{\gamma }}_{w}}=\frac{\tau_{w}}{\frac{{\dot{\gamma }}_{a}}{4}\cdot \left(3+\frac{{\mathrm{dS}(\tau }_{w})}{{\mathrm{dln}(\tau }_{w})}\right)}.$$

Here, the uncertainties of $\tau_{w}$, $a_{0}$, $a_{1}$ and $a_{2}$ propagate into the uncertainty of the viscosity $u_{\eta }$. The authors could not find a way to assess and incorporate the correlation between $u_{\tau_{w}}$ (Equation (18)) and the covariance $V_{\;a}$ (Equation (20)). We suggest the covariance

(23) $$V_{\tau_{w},\;a}=\left[\begin{array}{ccccc} u_{\tau_{w}}{}^{2} & 0 & 0 & 0 & 0\\ 0 & u_{a_{00}}{}^{2} & u_{a_{01}}{}^{2} & u_{a_{02}}{}^{2} & u_{a_{03}}{}^{2}\\ 0 & u_{a_{10}}{}^{2} & u_{a_{11}}{}^{2} & u_{a_{12}}{}^{2} & u_{a_{13}}{}^{2}\\ 0 & u_{a_{20}}{}^{2} & u_{a_{21}}{}^{2} & u_{a_{22}}{}^{2} & u_{a_{23}}{}^{2}\\ 0 & u_{a_{30}}{}^{2} & u_{a_{31}}{}^{2} & u_{a_{32}}{}^{2} & u_{a_{33}}{}^{2} \end{array}\right].$$

Neglecting the correlation terms in a PoU calculation is a common engineering approach. However, it causes, to some degree, an overprediction of the calculated uncertainty $u_{y}$ [30]. Applying the generalized PoU (Equation (13)) yields

(24) $$u_{\eta }=\sqrt{\left[\begin{array}{ccccc} \frac{\partial \eta }{\partial \tau_{w}} & \frac{\partial \eta }{\partial a_{0}} & \frac{\partial \eta }{\partial a_{1}} & \frac{\partial \eta }{\partial a_{2}} & \frac{\partial \eta }{\partial a_{3}}=0 \end{array}\right]\cdot V_{\tau_{w},\;a}\cdot \left[\begin{array}{c} \frac{\partial \eta }{\partial \tau_{w}}\\ \frac{\partial \eta }{\partial a_{0}}\\ \frac{\partial \eta }{\partial a_{1}}\\ \frac{\partial \eta }{\partial a_{2}}\\ \frac{\partial \eta }{\partial a_{3}}=0 \end{array}\right]}.$$

We used the Python library sympy [31] to perform the matrices operations of Equation (24) with inserted Equation (23) symbolically, which yields

(25) $$u_{\eta }=\frac{4\cdot \sqrt{\begin{array}{l} {\left(\frac{u_{\tau_{w}}}{\tau_{w}}\right)}^{2}\cdot {\left(3\cdot a_{0}\cdot \ln{\left(\tau_{w}\right)}^{2}\text{-}6\cdot a_{0}\cdot \ln\left(\tau_{w}\right)+2\cdot a_{1}\cdot \left(\ln\left(\tau_{w}\right)\text{-}1\right){+a}_{2}+3\right)}^{2}\\ +3\cdot \left(3\cdot \ln{\left(\tau_{w}\right)}^{2}\cdot u_{a_{00}}{}^{2}+2\cdot \ln\left(\tau_{w}\right)\cdot u_{a_{10}}{}^{2}{+u}_{a_{20}}{}^{2}\right)\cdot \ln(\tau_{w}{)}^{2}\\ +2\cdot \left(3\cdot \ln{\left(\tau_{w}\right)}^{2}\cdot u_{a_{01}}{}^{2}+2\cdot \ln\left(\tau_{w}\right)\cdot u_{a_{11}}{}^{2}{+u}_{a_{21}}{}^{2}\right)\cdot \ln\left(\tau_{w}\right)\\ +1\cdot \left(3\cdot \ln{\left(\tau_{w}\right)}^{2}\cdot u_{a_{02}}{}^{2}+2\cdot \ln\left(\tau_{w}\right)\cdot u_{a_{12}}{}^{2}{+u}_{a_{22}}{}^{2}\right) \end{array}}}{{\dot{\gamma }}_{a}\cdot {\left(3\cdot a_{0}\cdot \ln{\left(\tau_{w}\right)}^{2}+2\cdot a_{1}\cdot \ln\left(\tau_{w}\right){+a}_{2}+3\right)}^{2}}.$$

Readers should remember that Equation (25) does not explicitly include viscosity uncertainties due to fluctuations in the volumetric flow rates, temperatures, and counter-pressures. Such random errors are reflected in pressure variations and, consequently, larger uncertainties in the BCs and WRCs.

### 2.4. Cross-WLF Parameter Fitting

The parameters of the Cross-WLF viscosity model (Equation (2)) were fitted in two ways, using the Python scipy.optimize.curve_fit [11] routine:

- RSS fit: Equation (3) is minimized between the derived (measured) and the calculated (Cross-WLF) viscosities.
- WRSS fit: Equation (15) is minimized with the estimated uncertainties in the viscosity $u_{\eta }$ (Equation (25)) used as the weights.

For both fits, $D_{2}={413.15\;K=140\;{}^{\circ}C\sim T}_{g}$ (according to DSC measurement) and $A_{3}=51.6$ were chosen. The 5 remaining coefficients (n, $A_{1}$, $D_{1}$, $D_{3}$, and $\tau^{*}$) were free to vary within the lower and upper bounds of Table 1.

### 2.5. Flow Simulation of the Capillary Model

The capillary simulations were performed in Ansys^®^ 2022 R2 (Ansys Inc., Canonsburg, PA, USA): Ansys DesignModeler™ was utilized to create the capillary geometries as quarter models and Ansys Meshing™ for subsequent meshing. The simulation setups were performed in Polydata™, and the calculations were performed in Polyflow™. Finally, the computed pressures were retrieved through Ansys CFD-Post™.

**Figure 6:**
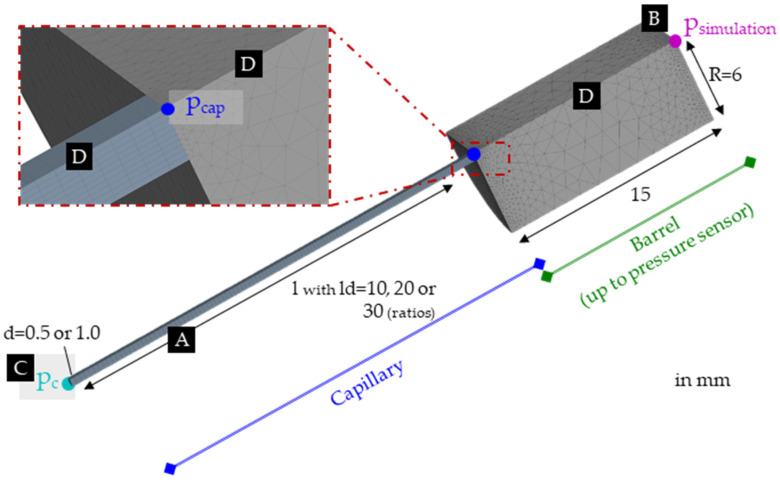
Setup for the simulation in Ansys Polyflow (shown here: d=1.0 mm and ld=30). Where (**A**) is the capillary, (**B**) is the pressure sensor’s location, (**C**) is the capillary’s outlet where the counter pressure is applied, and (**D**) is the part of the barrel modelled in the simulations.

Six models of the capillaries with diameters d=0.5 and 1 mm and ld-ratios 10, 20, and 30 (Figure 6A) were made. Consequently, all the in-practice measured HPCR settings (Table 2) could be simulated.

The HPCR barrel was modeled up to the pressure sensor position and specified as the inflow boundary with a constant temperature of (Figure 6B). Here, the investigated volumetric flow rates were assigned through an evolution scheme. This means that the highest flow rate ${\dot{V}}_{\max}$ (proportional to the highest corresponding shear rate given in Table 2) multiplied by a factor s was set for each capillary simulation. The factor was then varied, starting from $s_{0}=0.04$ to $s_{\mathrm{final}}=1,$ with a step size of Δs=0.02 for the 0.5 mm capillary with ${\dot{V}}_{\max}={0.6136\;\mathrm{cm}}^{3}/s$ (${\dot{\gamma }}_{a,\max}=50\;000\;1/s$). Likewise, for the 1.0 mm capillary, the factor was defined as $s_{0}=0.2$ and Δs=0.1 with ${\dot{V}}_{\max}={0.0982\;\mathrm{cm}}^{3}/s$ (${\dot{\gamma }}_{a,\max}=1500\;1/s$). Through this technique, by automatically adjusting the factor after each simulation, manual input was reduced. Finally, the flow rates matching the measurements were further evaluated to obtain the simulated pressures $p_{\mathrm{simulation}}$.

The end of the capillary (Figure 6C) was defined as a boundary condition upon which the examined counter-pressures $p_{c}$ could be imposed. Next, a zero-flow boundary and a no-heat flux boundary were assigned to the slicing planes of the quarter model (Figure 6D). A constant temperature (corresponding to the respective setting) and a zero-velocity boundary were assigned to the remaining surfaces (walls).

The capillary meshes were generated with a 0.25 mm sizing for the length and 10 divisions over the radius. In addition, the automatically assigned meshing settings were used for the barrel. The resulting meshes for the 0.5 mm capillaries featured 6 825 (ld=10), 8 321 (ld=20), and 10 795 (ld=30) nodes, and for the 1 mm capillaries 6 924 (ld=10), 10 558 (ld=20), and 13 862 (ld=30) nodes.

The governing equations for the non-isothermal simulations can be found in the Polyflow manual [32]. In contrast to reality, no viscous heating was calculated, as this assumption aligns with the measurements’ setup and derivations. The Ansys Polyflow Cross and WLF equations were modified through PMAT functions to account for the pressure dependency ($D_{3}\cdot p$ term) as defined in Equation (2). Through PMAT, some material properties can be dependent on local variables. Therefore, $D_{2}=1$ and $A_{3}=1$ were selected in Ansys Polyflow. Then, corresponding PMAT multiplications (a+b·p) were inserted, where a and b are constants, and p is the pressure. The PMAT values for $D_{2}$ were consequently ${a=A}_{3}$, and ${b=D}_{3}$ and for $A_{3}$, ${a=D}_{2}$, and ${b=D}_{3}$.

The simulations were made for both sets of Cross-WLF coefficients obtained by the RSS and WRSS fit, respectively. This led to 108 individual simulations that were analyzed concerning the pressure at the inflow $p_{\mathrm{simulation}}$ (Figure 6B) for comparison with the experiments.

The simulations were performed on a personal computer (12th Gen Intel^®^ Core™ i7-12700 2.1 GHz) with 16 GB RAM and a Windows 11 operating system with four threads, requiring about 500 s calculation time for each setup.

### 2.6. Procedure Set Up for a Virtual Material

In addition to the actual HPCR experiments (of the PC described in the sections above), a complete virtual HPCR experiment was simulated using the 1 mm capillary models and procedures described in Section 2.5. Here, a “virtual material” with arbitrarily chosen Cross-WLF viscosity coefficients stated in Table 3 was probed at the various temperatures and shear rates listed in Table 4.

This resulted in 90 simulated pressures, “repeated” 3 times (270 pressure results), and then subjected to normally distributed errors. Those errors in the pressures were applied using Microsoft Excel’s NORMINV(RAND(), $p_{\mathrm{simulation}}$, $p_{\mathrm{simulation}}\cdot \delta_{p}$) function. Herein, as stated in Table 4, larger relative errors of $\delta_{p}=0.2$ were used for the two lowest and highest and $\delta_{p}=0.1$ for the remaining shear rates. The reason was that, from experience, the measurements are the least reproducible at the lowest (slowly reacting pressure sensor, incompletely compressed melt) and highest (fast-depleting melt supply where the operator has to react quickly) shear rates.

Consequently, any possible infringements of the HPCR measurement assumptions and premises (see Section 1) could be ruled out, and the effects of random measurement errors could be tested exclusively. The deflected simulation pressures were then used to retrieve the corresponding viscosity values through the above-described data processing (BC, WRC) and viscosity PoU calculation.

Finally, the Cross-WLF parameters of the virtual material were re-obtained through the RSS and WRSS methods. For both fits, $D_{2}=413.15\;K$, $D_{3}=0\;K/\mathrm{Pa}$ (non-pressure dependent material), and $A_{3}=51.6$ were chosen. The 4 remaining coefficients (n, $A_{1}$, $D_{1}$, and $\tau^{*}$), were free to vary within the lower and upper bounds of Table 1.

(It should be remembered that, in practice, shear rates $\dot{\gamma }⪅\;100\;1/s$ can hardly be realized in a HPCR; however, such limitations do not exist in simulations. The wide shear rate range was deliberately chosen to obtain values in all three regions—Newtonian, transitioning, and shear thinning—of the viscosity curve.)

## 3. Results and Discussion

### 3.1. Viscosity Calculation

The viscosity data were derived using the procedure outlined in Section 2.3: linear BC plots were made according to Equation (16) to fit the pressure loss $p_{l}$ and parameter $b_{0}$ and to estimate the corresponding uncertainties $u_{p_{l}}$ and $u_{b_{0}}$, respectively. Hence, the wall shear stress $\tau_{w}{=b}_{0}/4$ (Equation (17)) with uncertainty $u_{\tau_{w}}{=u}_{b_{0}}/4$ (Equation (18)) were obtained. The (in total, 78) BC fits consisted of nine data points each (3 ld ratios with n=3 repeats) and yielded coefficients of determination $R_{\mathrm{BC}}^{2}$ ranging from 86.67% to 99.47% (median 97.11%).

**Figure 7:**
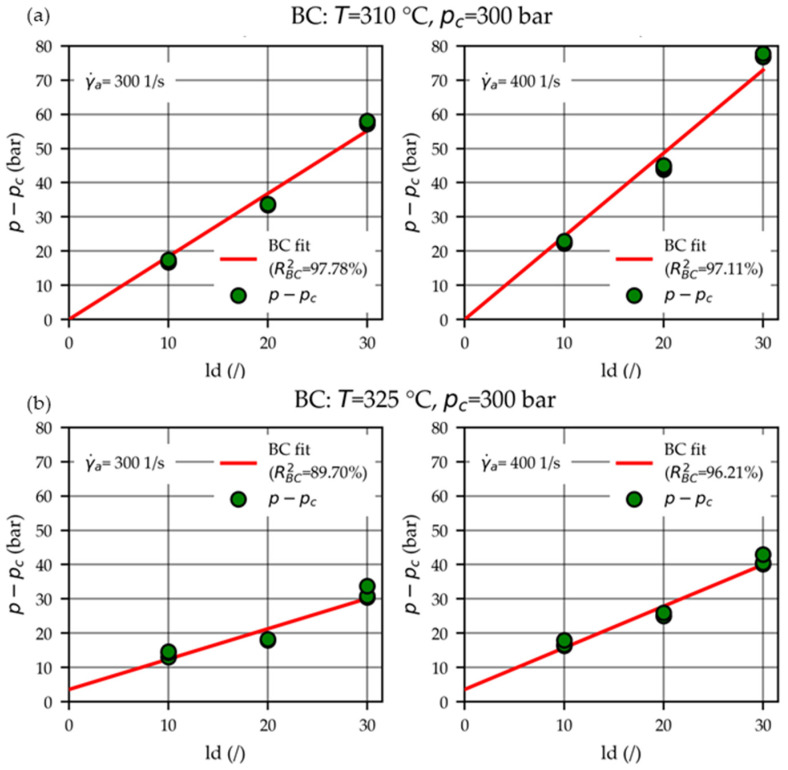
BC fits to obtain the wall shear stresses $\tau_{w}$ (Equation (17)), exemplarily shown for the measurements performed at the apparent shear rates ${\dot{\gamma }}_{a}=300$ and 400 1/s at temperatures T=310 °C (**a**) and 325 °C (**b**) when applying a counter-pressure of $p_{c}=300\;\mathrm{bar}$.

Four BC plots are exemplarily shown for the measurements performed at apparent shear rates ${\dot{\gamma }}_{a}=300$ and 400 1/s at temperatures T=310 °C and 325 °C when applying a counter-pressure of $p_{c}=300\;\mathrm{bar}$ in Figure 7a and Figure 7b, respectively.

Frequently pressure losses of $p_{l}=0$ were estimated, meaning that the imposed (lower) bound of a positive pressure loss $p_{l}\;\geq \;0$ was reached (see Figure 7a).

In the literature [14,15,22,23], the effects of shear heating and non-negligible pressure dependencies within the capillaries are frequently reported. A downward or upwards curvature in BC plots can indicate the presence of shear heating or pressure dependencies, respectively. However, in some cases, both effects may compensate for each other and become indistinguishable [22]. Quadratic BC polynomials [15,25] have been derived for pressure-dependent melts to avoid underestimating the pressure losses $p_{l}$. As visualized by the BCs in Figure 7, no curvature was apparent in our experiments, and consequently, quadratic polynomials were not utilized.

**Figure 8:**
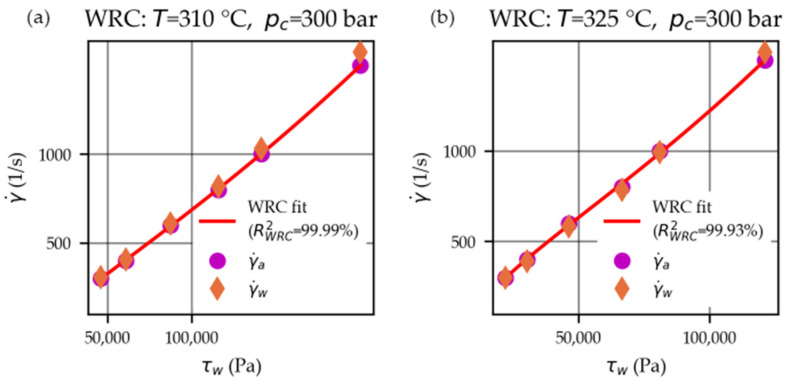
WRC fits to convert (Equation (21)) the apparent shear rates ${\dot{\gamma }}_{a}$ into the true shear rates ${\dot{\gamma }}_{w}$, exemplarily shown for the measurements performed at temperatures of T=310 °C (**a**) and 325 °C (**b**) when applying a counter-pressure of $p_{c}=300\;\mathrm{bar}$.

The (a total of 9) WRC fits of third degree yielded coefficients of determination $R_{\mathrm{WRC}}^{2}$ ranging from 99.80% to 99.99% (median 99.94%). Two WRC plots are exemplarily shown for the measurements performed at temperatures T=310 °C and 325 °C when applying a counter-pressure of $p_{c}=300\;\mathrm{bar}$ in Figure 8a and Figure 8b, respectively.

Consequently, the wall shear rates ${\dot{\gamma }}_{w}$ could be calculated using the estimated WRC parameters ($a_{0}$, $a_{1}$, and $a_{2}$) and Equation (21). Analogously, the (wall) viscosities η were obtained using Equation (22). Finally, the corresponding uncertainties $u_{\eta }$ were calculated using the derived Equation (25).

Table 5 shows an extract of the calculated HPCR measurement values at a counter-pressure of $p_{c}=300\;\mathrm{bar}$. The comparatively large uncertainties in the pressure loss terms ($u_{p_{l}}$) are related to the extrapolation to a die of ld=0 in the BCs. A more precise estimation of the pressure loss terms ($p_{l}$)—which were not further used in the presented analysis approach—should be possible by (additionally) measuring with an orifice die (“zero-length die”) [33,34].

### 3.2. Comparison of Cross-WLF Fit Using RSS and WRSS Minimization

Two Cross-WLF fits were made, as outlined in Section 2.4: one minimizing the residual sum of squares RSS (Equation (3)) and one minimizing the weighted residual sum of squares WRSS (Equation (15)), and therefore considering the derived uncertainties in the viscosity $u_{\eta }$ (Equation (25)). The corresponding RSS and WRSS coefficients are given in Table 6.

**Figure 9:**
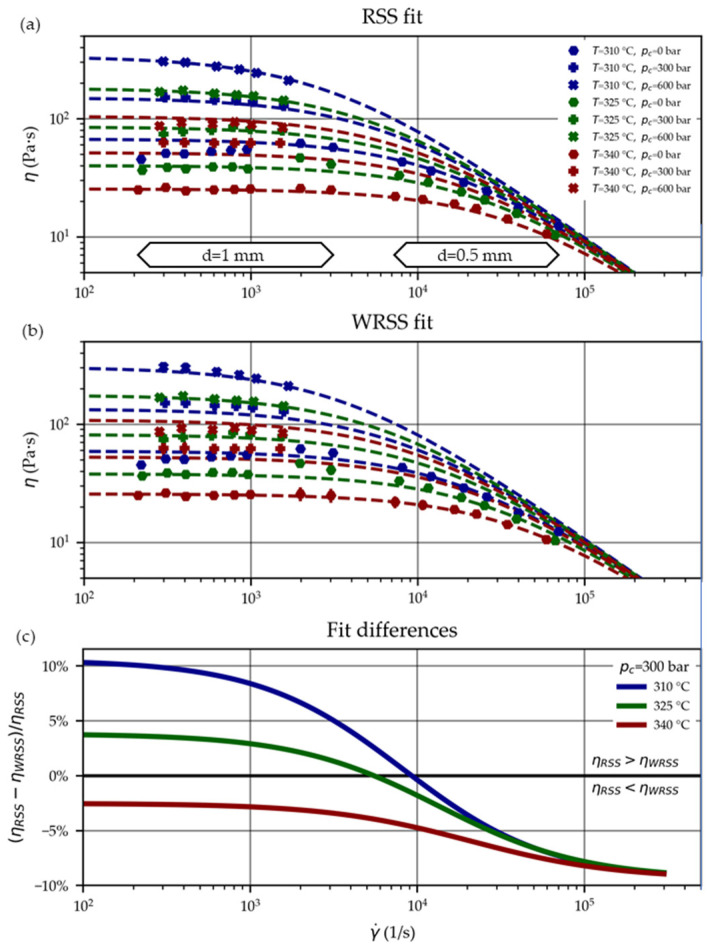
Viscosity curves of the PC Lexan OQ1028 based on the Cross-WLF parameter fit RSS (**a**) and WRSS (**b**) with markers designating the underlying experimental values. Their differences in percentage (at a pressure of p=300 bar) are visualized in (**c**).

Figure 9a,b illustrate the RSS and WRSS fit and the measured viscosity values (Section 3.1). Figure 9c then contrasts the fits by plotting their difference in percentage (at a pressure of p=300 bar), showing differences within the range of about ±10%. Those differences can be further reflected when calculating the RSS according to Equation (3), which are ${\mathrm{RSS}}_{\mathrm{RSS}\;\mathrm{fit}}=3352\;(\mathrm{Pa}\cdot {s)}^{2}$—where it was used as the minimization function—and ${\mathrm{RSS}}_{\mathrm{WRSS}\;\mathrm{fit}}=6655\;(\mathrm{Pa}\cdot {s)}^{2}$. The latter is ~2 times larger, demonstrating the impact of the introduced weights $1/u_{\eta }{}^{2}$ to the WRSS fit.

### 3.3. Comparison of Fits Regarding Simulation Results

**Figure 10:**
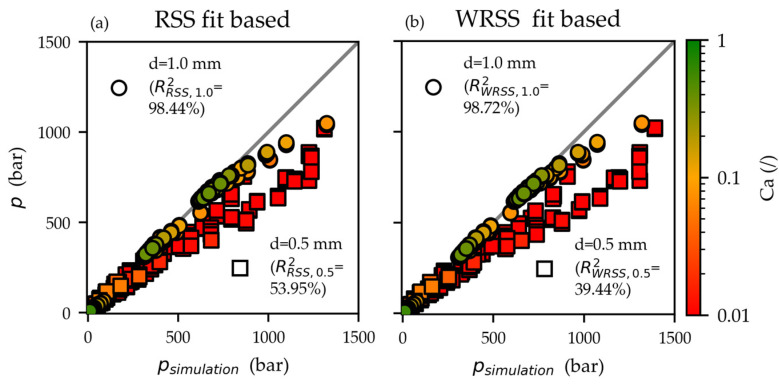
Contrasting of measured HPCR pressures p with the simulated pressures $p_{\mathrm{simulation}}$ based on the Cross-WLF parameter fits RSS (**a**) and WRSS (**b**). A better agreement exists between measured and simulated values for the d=1 mm capillaries (circular symbols) than the narrower d=0.5 mm capillaries (square symbols). Low Cameron numbers Ca (Equation (12)) suggest non-isothermal conditions. It could explain the overestimation of the pressures in the isothermal simulations.

The simulated pressures $p_{\mathrm{simulation}}$ based on the RSS and WRSS fits are compared against the measured pressures p (single values) in Figure 10a and Figure 10b, respectively. The d=1.0 mm capillaries (circular symbol) simulations match noticeably better than those of the narrower d=0.5 mm capillaries (square symbols).

The Cameron numbers Ca were calculated according to Equation (12) with the material properties in Section 2.1 and are represented in Figure 10 by the color bar. The low Ca ≈ 0.01 numbers for the d=0.5 mm capillaries indicate non-isothermal conditions [27]. This could explain the overestimation of the pressures in the isothermal simulations. In reality, substantial dissipative heating causes an increase in the temperature of the melt. It decreases the viscosity of the melt resulting in a lower observed pressure drop (compared to isothermal flow conditions).

Consequently, the HPCR evaluation requirement of the isothermal flow is violated [13,14]. As a result, the derived viscosities (for the selected and thus presumed temperature T) are underestimated. In the Cross-WLF fits (RSS and WRSS), this is also reflected in the unusually low power law indexes n=0 (Table 6), feigning an excessively high degree of shear thinning.

The WRSS fit predicts higher viscosities than the RSS fit at higher shear rates, as shown in Figure 9c. As a result, the simulated pressures based on the WRSS fit overestimated the measured pressures even more considerably ($R_{\mathrm{RSS},\;0.5}^{2}=53.95\%$ vs. $R_{\mathrm{WRSS},\;0.5}^{2}=39.44\%$). Ultimately—due to the issues of non-isothermal flow conditions in the d=0.5 mm capillaries—it cannot be determined which Cross-WLF fit (RSS or WRSS) more accurately describes the viscosity at high shear rates. However, both fits most likely lack a satisfying degree of accuracy.

**Figure 11:**
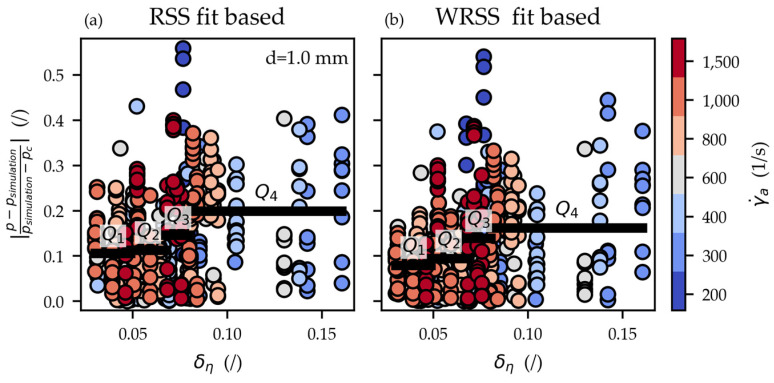
Relative deviations of the measured and simulated pressure (Equation (26)) using the RSS fit (**a**) and WRSS fit (**b**) plotted vs. the relative viscosity uncertainties $\delta_{\eta }=\;u_{\eta }/\eta$ for the d=1 mm capillaries. The data were split into even quartiles ($Q_{1}$–$Q_{4}$) with the black horizontal line indicating the corresponding mean deviation. Especially $Q_{1}$ of the WRSS simulations is smaller compared to that of the RSS simulations. This indicates that the WRSS fit-based simulation delivers more precise predictions for those pressures that were measured more reproducible.

The simulations for the d=1.0 mm capillaries yield almost equivalent coefficients of determination when using the RSS ($R_{\mathrm{RSS},\;1.0}^{2}=98.44\%$) and WRSS ($R_{\mathrm{WRSS},\;1.0}^{2}=98.72\%$) based Cross-WLF coefficients. Those simulations were further scrutinized by plotting the deviation

(26) $$\left|\frac{{p\text{-}p}_{\mathrm{simulation}}}{p_{\mathrm{simulation}}{\text{-}p}_{c}}\right|$$

of the simulation results vs. the relative viscosity uncertainty $\delta_{\eta }{=u}_{\eta }/\eta$ for the RSS and WRSS fit-based simulations in Figure 11a and Figure 11b, respectively. Here, the simulation deviation Equation (26) measures how well the simulated and measured pressures correspond (*y* axis in Figure 11). The relative viscosity uncertainty $u_{\eta }/\eta$, in contrast, is a measure of how reproducible the viscosities were measured (*x* axis in Figure 11).

The deviations of the WRSS fit-based simulations are, on average, 16% smaller than those of the RSS fit. The data in Figure 11 were further split into even quartiles ($Q_{1}$–$Q_{4}$) with the black horizontal line indicating the corresponding mean deviations. The WRSS fit-based quartiles $Q_{1}$, $Q_{2}$, $Q_{3}$, and $Q_{4}$ are 25%, 17%, 5%, and 19% smaller than their RSS fit-based counterparts. Seemingly viscosities that were measured with a higher degree of certainty are depicted better by the WRSS fit.

### 3.4. Method Scrutinization through the Virtual Material

The “virtual material” viscosity data were derived using the same procedure as for the PC, with the exception that it was assumed to be non-pressure dependent ($D_{3}=0\;K/\mathrm{Pa}$). The re-fitted Cross-WLF parameters using the simulated pressures offset with normally distributed errors are given in Table 7.

**Figure 12:**
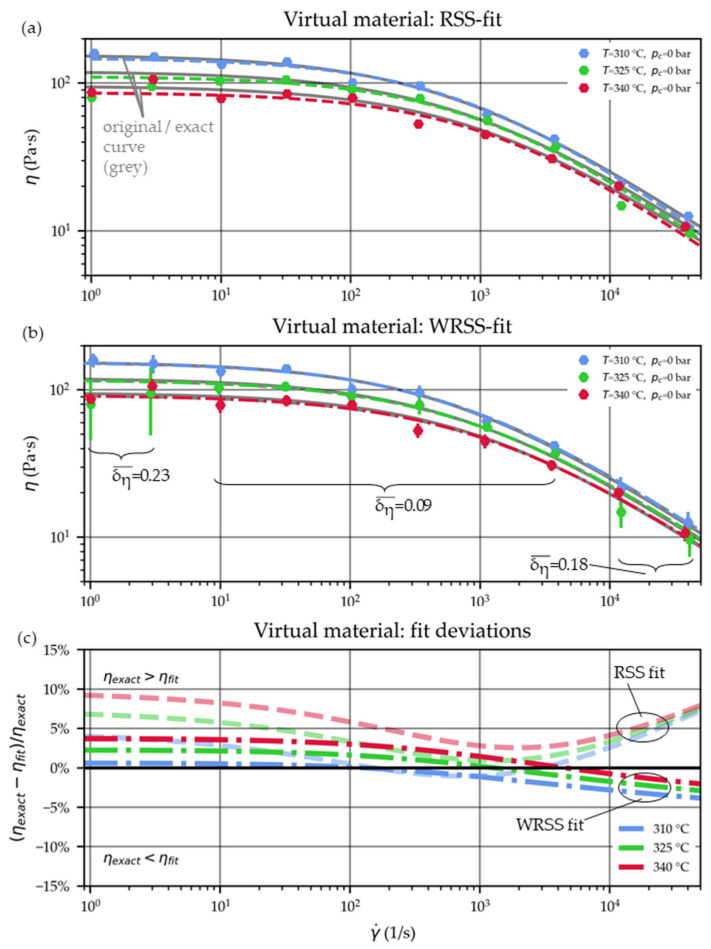
The Cross-WLF viscosity curves of the virtual material are based on parameter optimizations using the RSS (**a**) and the WRSS (**b**) approaches. The initial (exact) curves using the parameters of Table 3 are plotted in grey. The percentage differences from this curve are visualized in (**c**) as dashed lines for the RSS fit and dash-dotted lines for the WRSS fit.

Figure 12a plots the corresponding derived viscosity values and the Cross-WLF viscosity curves for the RSS fit. The exact curves (based on the parameters of Table 3) are additionally underlaid in grey. Similarly, the WRSS fit-based curves are shown in Figure 12b, where the error bars denote the calculated viscosity uncertainties.

Finally, the deviations from the original (hence “exact”) parameters of Table 3 are drawn in Figure 12c.

The PoU calculation showed larger viscosity uncertainties for the low and high shear rate domains, depicted by the error bars in Figure 12b. This is in line with expectation: here, higher relative pressure errors were imposed ($\delta_{p}=0.2$ compared to $\delta_{p}=0.1$ for the pressures at the other shear rates). In this particular example, the averaged relative viscosity uncertainty was $\bar{\delta_{\eta }\;}=0.23$ for the low apparent shear rates of 1 and 3 1/s, $\bar{\delta_{\eta }\;}=0.18$ for the high shear rates of 10,000 and 31,623 1/s and $\bar{\delta_{\eta }\;}=0.09$ for the remaining shear rates in between.

Furthermore, the WRSS fit ($\left|\Delta \eta_{\mathrm{WRSS}}\right|\;<\;5\%$) is less adversely affected by those faulty data points than the RSS fit ($\left|\Delta \eta_{\mathrm{RSS}}\right|\;<\;10\%$). For context, using the simulated pressure result without subjection to the normally distributed errors restore the initial Cross-WLF parameters with viscosity deviations of less than 1%.

## 4. Conclusions

Measuring the shear viscosity η of polymers under relevant processing conditions such as elevated shear rates $\dot{\gamma }$, temperatures T, and pressure p is an extensive effort. For this, high-pressure capillary rheometers (HPCRs) are frequently used, where the pressure required to push a melt at a defined volumetric flow rate $\dot{V}$ through a narrow capillary (round die) is recorded. Multiple premises, such as isothermal conditions, must be fulfilled during those experiments [13,14]. Furthermore, Bagley corrections (BCs) [16] and Weissenberg–Rabinowitsch corrections (WRCs) [17] must be performed to convert the measured pressures into viscosity values, which involves parameter fitting.

Random errors in the (repeatedly) measured pressures and ambiguities in the corrections (BCs, WRCs) affect the accuracy of the derived viscosities. Those might originate, for instance, from insufficiently melted and manually compressed granulates, not fully developed steady-state flow conditions, slowly responding pressure signals, and fluctuations in the applied counter-pressures. Therefore, in the present work, a propagation of uncertainty (PoU) calculation was performed along the HPCR data evaluation. Consequently, the uncertainties in the viscosities originating from the random variations in the measurements could be quantified.

The procedure was experimentally demonstrated for a polycarbonate (PC) melt measured in a HPCR. Capillaries of a diameter of d=1.0 mm were used for shear rates below $\dot{\gamma }\;<\;1500\;1/s$, and d=0.5 mm capillaries were used for the shear rates up to 5000 1/s.

Next, the measured viscosities of the PC were used to fit the Cross-WLF viscosity model [4,5,6,7,8] parameters through the traditional residual sum of squares (RSS) minimization approach. In addition, a weighted residual sum of squares (WRSS) [10,11] fit was made, which incorporated the derived viscosity uncertainties $u_{\eta }$. The motivation was that, by doing so, individual poorly measured (lower reproducibility) viscosity values should less negatively influence the overall fit quality. The two fits showed differences between ±10% (depending on the observed shear rate, temperature, and pressure level).

The HPCR measurements were studied in numerical simulations with both fits. Here, the WRSS fit-based simulations of the d=1.0 mm capillaries matched the measured pressures on average 16% closer. Thereby, in particular, measurements with smaller relative viscosity uncertainties $u_{\eta }/\eta$ were more accurately predicted with the WRSS fits. Apparently, considering the uncertainties led to more precise coefficients. For the narrower d=0.5 mm capillaries, severe non-isothermal conditions (Cameron numbers Ca⪅0.01 [27]) and thus violations of the HPCR evaluation prerequisites were detected. This systematic error was reflected in substantial deviations between the measured and simulated pressures $p_{\mathrm{simulation}}$ for both fits ($R_{\mathrm{RSS},\;0.5\;}^{2}=53.95\%$ vs. $R_{\mathrm{WRSS},\;0.5}^{2}=39.44\%$).

In addition, the HPCR measurement simulations of a ”virtual material” with arbitrary but known Cross-WLF parameters were made, and the simulated pressures were deliberately deflected by a normally distributed error. Consequently, systematic errors could be ruled out in a further comparison of the re-fitted Cross-WLF parameters using the RSS and WRSS approaches. The WRSS fit, which took advantage of the calculated viscosity uncertainties (PoU), could better restore the initial parameters.

With today’s computational possibilities, incorporating PoU calculations into the viscosity derivation is straightforward and was demonstrated in the present work. This allows more meaningful viscosity graphs to be plotted with error bars (standard deviations) indicating the repeatability/reliability of the underlying measurements.

Furthermore, the hence-derived viscosity uncertainties can be utilized for WRSS parameter optimizations of viscosity models such as the Cross-WLF model. Hence, it is possible to account for unequal precision in the calculated viscosity values to improve the fit quality. This can be useful for computational fluid dynamics, heavily relying on accurately described materials.

However, it has also been shown that HPCR measurement requirements, such as isothermal flow conditions, must be strictly adhered to in order to prevent systematic errors from biasing the measurement and fitting results.

## Figures and Tables

**Table 1:** Cross-WLF viscosity model (Equation (2)) parameters, including limits as imposed in the simulation software Autodesk Moldflow Insight [4].

Symbol	Unit	Designation	Lower Bound	Upper Bound
$D_{2}$	K	Reference temperature (frequently: $D_{2}{=T}_{g}$)	0	$10^{3}$
$D_{1}$	Pa∙s	Zero-shear viscosity at ${T=D}_{2}$	0	-
$\tau^{*}$	Pa	Critical stress level at the transition to shear thinning	0	$10^{9}$
n	/	Power law index slope in the shear thinning domain	0	1
$A_{1}$	/	WLF parameter	0	$40^{4}$
$A_{3}$	K	WLF parameter (frequently: 51.6 K)	0	$20^{4}$
$D_{3}$	K/Pa	Linear pressure dependence	0	$10^{-5}$

**Table 2:** Experimental plan of the HPCR viscosity measurements of the PC Lexan OQ1028.

Temperature T (°C)	Counter-Pressure $p_{c}$ (bar)	Capillary Configuration	Apparent Shear Rate ${\dot{\gamma }}_{a}$ (1/s)
310, 325, 340	0	d=1 mm, ld=10, 20, 30	200, 300, 400, 600, 800, 1000
		d=0.5 mm, ld=10, 20, 30	2000, 3000, 7000, 10,000, 15,000, 20,000, 30,000, 50,000
	300, 600	d=1 mm, ld=10, 20, 30	300, 400, 600, 800, 1000, 1500

**Table 3:** Cross-WLF viscosity (Equation (2)) coefficients for the virtual material.

$D_{1}$ (Pa·s)	$\tau^{*}$ (Pa)	n (/)	$A_{1}$ (/)	$D_{3}$ (K/Pa)	$A_{3}$ (K)	$D_{2}$ (K)
${1.0\;\times \;10}^{8}$	${1.0\;\times \;10}^{5}$	0.40	17.44	0	51.6	413.5

**Table 4:** The experimental plan for the simulated HPCR measurements of the virtual material. This resulted in 90 simulated pressures, “repeated” 3 times (270 pressure results), and then subjected to normally distributed errors.

Temperature T (°C)	Capillary Configuration	Apparent Shear Rate ${\dot{\gamma }}_{a}$ (1/s)	Subjected Relative Error $\delta_{p}$ (/)
310, 325, 340	d=1 mm, ld=10, 20, 30	1, 3,	0.2
		10, 32, 100, 316, 1000, 3162,	0.1
		10,000, 31,623	0.2

**Table 5:** Extract from the calculated HPCR measurement values at a counter-pressure of $p_{c}=300\;\mathrm{bar}$ for two temperatures (T) at the investigated apparent shear rates (${\dot{\gamma }}_{a}$). The pressure loss terms ($p_{l}\pm u_{p_{l}}$) and wall shear stresses ($\tau_{w}\pm u_{\tau_{w}}$) were obtained through linear BC plots with respective coefficients of determination ($R_{\mathrm{BC}}^{2}$). Next, the true wall shear rates (${\dot{\gamma }}_{w}$) were obtained through third-degree WRC with respective coefficients of determination ($R_{\mathrm{WRC}}^{2}$). Finally, the viscosities (η) were calculated using Equation (22) and their respective uncertainties ($u_{\eta }$) using Equation (25). The relative uncertainties in the viscosities are given in the $\delta_{\eta }{=u}_{\eta }/\eta$ column.

T (°C)	${\dot{\gamma }}_{a}$ (1/s)	$p_{l}\pm u_{p_{l}}$ (bar)	$\tau_{w}\pm u_{\tau_{w}}$ (bar)	$R_{BC}^{2}$ (/)	${\dot{\gamma }}_{w}$ (1/s)	$R_{WRC}^{2}$ (/)	${\eta \pm u}_{\eta }$ (Pa·s)	$\delta_{\eta }$ (/)
310	300	0.00 ± 2.49	0.46 ± 0.03	97.78%	306.76	99.99%	149.92 ± 10.08	0.07
	400	0.00 ± 3.54	0.61 ± 0.04	97.11%	406.28		149.58 ± 10.35	0.07
	600	0.00 ± 9.86	0.87 ± 0.11	90.47%	609.08		143.57 ± 18.67	0.13
	800	0.00 ± 8.25	1.16 ± 0.10	95.83%	817.02		142.12 ± 11.44	0.08
	1000	0.00 ± 10.15	1.42 ± 0.12	95.77%	1029.11		137.90 ± 11.00	0.08
	1500	0.00 ± 13.34	2.01 ± 0.15	96.77%	1574.33		127.71 ± 9.36	0.07
325	300	3.57 ± 2.45	0.22 ± 0.03	89.70%	299.39	99.93%	73.89 ± 10.51	0.14
	400	3.59 ± 1.96	0.30 ± 0.02	96.21%	391.78		77.37 ± 6.12	0.08
	600	3.70 ± 2.54	0.46 ± 0.03	97.26%	583.24		79.28 ± 5.08	0.06
	800	0.49 ± 3.85	0.67 ± 0.04	96.97%	785.38		84.78 ± 5.47	0.06
	1000	2.28 ± 3.74	0.81 ± 0.04	98.04%	993.05		81.55 ± 4.12	0.05
	1500	0.77 ± 4.01	1.21 ± 0.05	98.99%	1544.96		78.52 ± 3.62	0.05

**Table 6:** Cross-WLF viscosity model (Equation (2)) coefficients for the PC Lexan OQ 1028 in which either the RSS (Equation (3)) or the WRSS (Equation (15)) was used as the minimization function. For both fits, the following fixed parameters were selected $D_{2}=413.15\;K$ (approximately $T_{g}$ of PC), and $A_{3\;}=51.6\;K$.

Fit Type	$D_{1}$ (Pa·s)	$\tau^{*}$ (Pa)	n (/)	$A_{1}$ (/)	$D_{3}$ (K/Pa)
RSS fit	$3.1\times 10^{13}$	$1.0\times 10^{6}$	0	35.0	$1.7\times 10^{-7}$
WRSS fit	$6.0\times 10^{11}$	$1.1\times 10^{6}$	0	30.0	$2.0\times 10^{-7}$

**Table 7:** Cross-WLF viscosity model coefficients for the virtual material in which either the RSS (Equation (3)) or the WRSS (Equation (15)) was used as the minimization function. For both fits, the following fixed parameters were selected $D_{2}=413.15\;K$, $A_{3}=51.6\;K$, and $D_{3}=0\;K/\mathrm{Pa}$ (the original parameters upon which the simulations were based are given in Table 3).

Fit Type	$D_{1}$ (Pa·s)	$\tau^{*}$ (Pa)	n (/)	$A_{1}$ (/)
Original	${1.0\;\times \;10}^{8}$	${1.0\;\times \;10}^{5}$	0.40	17.44
RSS fit	${4.34\;\times \;10}^{8}$	${1.15\;\times \;10}^{5}$	0.37	19.41
WRSS fit	${2.40\;\times \;10}^{8}$	${1.04\;\times \;10}^{5}$	0.40	18.59

## Data Availability

The data presented in this study are available upon request from the corresponding author.
